# Nano Filling Effect of Nonmeat Protein Emulsion on the Rheological Property of Myofibrillar Protein Gel

**DOI:** 10.3390/foods11050629

**Published:** 2022-02-22

**Authors:** Ruying Cai, Zongyun Yang, Zhen Li, Peng Wang, Minyi Han, Xinglian Xu

**Affiliations:** Key Laboratory of Meat Processing and Quality Control, Ministry of Education, College of Food Science and Technology, Nanjing Agricultural University, Nanjing 210095, China; jncaianna@163.com (R.C.); 2021208021@stu.njau.edu.cn (Z.Y.); 2018808149@njau.edu.cn (Z.L.); myhan@njau.edu.cn (M.H.); xlxus@njau.edu.cn (X.X.)

**Keywords:** myofibrillar protein, nano-emulsion, rheological property, active interaction

## Abstract

Incorporation of vegetable oils through pre-emulsification has received notable attention for delivering polyunsaturated fatty acids to emulsified-type meat products. The two important influencing factors of the rheological property of composite myofibrillar protein (MP) gel are emulsion droplet size and active or inactive interaction between interface and meat proteins. Incorporation of nonmeat protein emulsion (2% protein (*w*/*w*), egg-white protein isolate (EPI), porcine plasma protein (PPP), or sodium caseinate (SC)) with different droplet sizes (nano or macro) to a model of 2% MP gel was investigated in this research. The results of drop size measurement showed that 15,000 psi homogenizing could decrease the diameter of emulsion drop from macro- to nanoscale in the range of 324.4–734.5 nm. Active fillers (PPP and EPI emulsions) with nanodroplet size did not influence the viscosity of emulsion-filled composite cold sols but caused positive filling effects on the MP gel matrix after heating, as evidenced by the density microstructure. PPP and EPI nano-emulsion-filled composite MP had a significant high storage modulus enforcement effect, which reached nearly eight times those of other treatments (*p* < 0.05). Similarly, the results of thermal scanning rheology and a large-deformation mechanical test showed that PPP and EPI emulsions with nanoscale droplets, other than macroscale, had the highest gel strength of heat-induced emulsion-filled composite MP gel (*p* < 0.05). Overall, these findings will be helpful for selecting the correct pre-emulsified protein and designing the textural properties of foods.

## 1. Introduction

In recent years, consumers have paid close attention to the harmful effects that are led by saturated fats in their daily diet. Therefore, an important consumer-driven trend of buying healthy meat derivatives exists, in which fat content is reduced or animal fat is replaced using vegetable oil with the recommendations [1,2].

Emulsions are droplets of one fluid stabilized by a surfactant dispersed in a second immiscible fluid [3]. In the typical emulsified gel meat products, fat globules are stabilized by a membrane coating made largely of salt-soluble myofibrillar protein (MP), which can localize fat globules and is the formation of a cohesive protein matrix with the capability of holding fat and water physically [4]. However, the stability mechanism of oil drop differs for products adopting the pre-emulsification process. Such products can be achieved by the pre-emulsification process, in which nonmeat proteins are used as pre-emulsifier, and this pre-emulsified emulsion is filled into the meat batter. Therefore, later types of emulsified gel meat products can be considered soft solid colloids; nonmeat protein emulsion droplets in this type are incorporated into MP matrices. Except for the strength of the matrix of MP, the mechanical properties of these emulsion-filled gels depend on the property of emulsion droplets, such as size, volume fraction, and distribution [5]. Nano-emulsion is also named as mini or submicron emulsion, and the droplet sizes of the gels fall in the range of several hundred nanometers, such as 100–1000 nm [6] and 20–500 nm [7]. In recent years, most studies on nano-emulsion have focused on kinetic stability and delivery property [8,9,10,11,12]. Micron-sized glass or wax particle increases textural properties when it is filled into a comminuted meat matrix [13,14]. Therefore, the effect of nano-sized emulsion droplets on the mechanical properties of emulsion-filled food gels should be investigated on the basis of previous studies on the texture-modifying effects of micrometer-scale oil droplets on emulsion-filled gels [15].

Nonmeat protein type has an important influence on the mechanical properties of emulsion filling gels due to possible interactions between nonmeat protein and MP. The characteristics of fillers should be understood because the interaction between proteins contributes to the formation of the gel. Filler particles (or droplets), with different effects on network rheology, can be classified as either “active” or “inactive” [16]. Active fillers can intimately incorporate with the gel matrix; thus, they not only can fill crevices or pores but can also increase the gel strength [17]. On the contrary, inactive fillers can weaken the gel strength. Although sodium caseinate (SC), porcine plasma protein (PPP), and egg-white protein isolate (EPI) are all potentially introduced as an animal fat replacer in meat products [18,19,20,21], the “active” or “inactive” filling effects of the three proteins should be clarified.

To the best of our knowledge, experimental and theoretical studies on the combination of nano-emulsion and interfacial protein types are rare. This study aimed to determine the effects of three nonmeat protein (PPP, EPI, and SC) emulsions with different droplet sizes on the rheological properties and filling effects of model MP-emulsified composite gels. The results of this research will help elucidate the regulation rule of heat-induced meat protein gelation through the aspects of protein interaction and varying emulsion droplet diameter scales. It can also help provide a manipulating method utilizing an emulsion gel system for pharmaceutical, cosmetic, and food applications.

## 2. Materials and Methods

### 2.1. Materials

Chicken breasts were purchased from a local supermarket (Nanjing, China). Spray-dried PPP (protein content ≥ 78%) was purchased from Baodi Agricultural Science and Technology Co., Ltd. (Tianjin, China). EPI (protein content ≥ 81%) was donated by Mengniu Dairy Company Co., Ltd. (Anhui, China). SC (protein content ≥ 82%) was purchased from BMD Labservice Co., Ltd. (Jiangsu, China). Jinlongyu soybean oil produced by Yihai Kerry Food Company (Shanghai, China) was purchased from a local supermarket. The soybean oil contained 16% saturated fatty acid, 25% monounsaturated fatty acid, and 59% polyunsaturated fat. According to the manufacturer’s declaration, the soybean oil contained 16% saturated fatty acid, 25% monounsaturated fatty acid, and 59% polyunsaturated fatty acids.

### 2.2. Preparation of MP

MP was extracted following the procedure described by Han [22] with minor modifications, according to Zhao [23]. Trimmed, minced, weighed chicken breast was washed by four volumes of ice-cold standard salt solution (100 mmol L^−1^ KCl, 20 mmol L^−1^ K_2_HPO_4_/KH_2_PO_4_, 2 mmol L^−1^ MgCl_2_, 1 mmol L^−1^ EGTA, 1 mmol L^−1^ NaN_3_, pH 7.0, 4 °C) and homogenized for 30 s at 7000 r/min (repeated twice) using an Ultra Turrax T25 BASIS. The homogenates were filtrated before centrifuging (2000× *g*, 10 min, 4 °C). The myofibrillar pellet was collected between washes. The aforementioned steps were repeated two or three times to obtain high-quantity MP. The pellet was then homogenized with four volumes of 0.1 mmol L^−1^ KCl and centrifuged (2000× *g*, 10 min, 4 °C) for four times. After the last centrifugation, the pellet was collected as pure MP and immediately stored at 4 °C. All extraction steps were operated at 0 °C–4 °C. The protein concentration of pure MP was determined by the biuret method, and bovine serum albumin was used as the standard.

### 2.3. Preparation of Emulsion

Oil/water (O/W) emulsions (30% *w*/*w*) were prepared in phosphate buffer (0.6 M NaCl, 20 mM Na_2_HPO_4_/NaH_2_PO_4_, pH 6.25) to yield protein: oil: phosphate buffer ratio of 2%:30%:68% (by weight) [24]. The proteins, which contained MP, PPP, EPI, and SC, were dissolved into the phosphate buffer and stirred overnight to allow complete protein hydration. Macro-emulsion was prepared by homogenizing the protein solution two times at 8000 rpm with soybean oil for 30 s using an Ultra Turrax T25 BASIS (IKA, Staufen im Breisgau, Germany). Nano-emulsion was made by homogenizing the macro-emulsion (except MP macro-emulsion) with a high-pressure homogenizer (Mini DeBee, Bee International, South Easton, MA, USA) at 15,000 psi for two passes followed by centrifugation (1000× *g*, 5 min, 4 °C) to remove air bubbles. The MP macro-emulsion group was regarded as the control during the whole experiment.

### 2.4. Preparation of MP-Emulsified Soybean Oil Composite Sols

The MP-emulsified soybean oil composite sols were made by mixing different emulsions, as described above, with MP in 0.6 M NaCl and 20 mM phosphate buffer (pH 6.25) and then by homogenizing two times for 30 s at room temperature with an Ultra Turrax T25 BASIS (IKA, Staufen im Breisgau, Germany) at a speed of 7000 rpm. In the mixtures, the final protein concentration of emulsion and MP was 2% after combination.

### 2.5. Preparation of Heat-Induced Gelation

Prior to gelation, protein–soybean oil composite sols were centrifuged at 2000× *g* for 10 min to remove the bubbles. A total of 10 g of the sols was packed and placed into glass vials. Gels were made by heating from 20 °C to 80 °C at a rate of 2 °C/min in a linear-heating water bath (ZKSY-600, Keer Co., Ltd., Nanjing, China). After the target temperature (80 °C) was reached, the gels were insulated in the water bath at 80 °C for 10 min. Then, they were collected from the water bath and stored at 4 °C overnight.

### 2.6. Measurement of Drop Size of Emulsion

#### 2.6.1. Measurement of Drop Size of Macro-Emulsion

The droplet size distribution of the macro-emulsions was determined by dynamic light scattering (DLS) using a Malvern Mastersizer 3000 (Malvern Instruments Ltd., Worcestershire, UK) connected to Hydro LV (Malvern Instruments Ltd., Worcestershire, UK), which is a large volume automatic wet dispersion unit. The refractive indices of water and the dispersed phase (soy oil) were set to 1.330 and 1.456. The absorbance was adjusted to 0.001. The volume mean diameter (*D*_4,3_) was taken to be the mean droplet diameter, which is mathematically expressed as:

(1) $$D_{4,3}=\frac{\sum D_{i}^{4}n_{i}}{\sum D_{i}^{3}n_{i}^{\prime }}$$

where *n_i_* means the total number of droplets with diameter *D_i_*.

All the experiments were performed 5 times and averaged.

#### 2.6.2. Measurement of Drop Size of Nano-Emulsion

A Zetasizer Nano ZS 90 (Malvern Instruments, Worcestershire, UK), which employed a DLS technique, was used at 25 ± 0.1 °C to evaluate the droplet diameter of nano-emulsions. Emulsions were diluted with water to 0.2 mg/g and placed into a cuvette. Mean particle diameter (z-average diameter), size distribution, and polydispersity index were used to represent the droplet diameter. All the experiments were performed 5 times and averaged.

### 2.7. Measurement of Interfacial Tension

The dynamic interfacial tension of proteins at the soybean O/W interface was measured using the pendent drop method with an OCA 25 video-based optical contact angle meter (Dataphysics Instruments GmbH, Filderstadt Germany). The soybean oil was placed in a syringe and settled at room temperature. The oil droplets (15 μL) were pushed into a cuvette with the protein solution (0.3 mg/mL) by the electric injection unit, and droplets were formed on the tip [25]. The drop shape was recorded by a charge-coupled device system, and the interfacial tension was calculated automatically by SCA 20 software (Dataphysics Instruments GmbH, Filderstadt, Germany). The data reported were obtained from at least five replicates.

### 2.8. Viscosity and Heat Scanning Rheological Measurements

Dynamic rheological measurements include viscoelastic properties and viscosity. Both measurements were conducted using MP-emulsified soybean oil composite sols, as described above, by a rheometer (Physica MCR301, Anton Paar Corporation, Graz, Austria), which is equipped with a Peltier plate temperature control unit (P-PTD 200) (Physica MCR301, Anton Paar Corporation, Graz, Austria).

Viscosity was measured by rotational shear between the two 50 mm-diameter parallel plates at 25 °C with a gap of 1 mm as a function of increasing shear rate from 0.1 s^−1^ to 1000 s^−1^ with a total of 330 s.

Viscoelastic properties were used to examine the dynamic formation of a protein network during gelation [24]. Protein sols were heated from 20 °C to 80 °C at a rate of 2 °C/min [26]. During monitoring of gelation, composite sols were loaded between two parallel plates with a 1 mm gap and at 0.1 Hz within a strain of 1%, according to the references [26,27,28,29,30]. Changes in the storage modulus (G′) and loss modulus (G″) were recorded continuously. A layer of light mineral oil was used to cover the samples for preventing water loss. The experiments were performed in triplicate. 

### 2.9. Gel Strength Measurement

Before the gel strength measurement, heat-induced emulsion-filled gels were equilibrated at room temperature for 30 min. Gel strength was measured using a TA-XT plus texture analyzer (Stable Micro Systems Co. Ltd., Surrey, UK) [26]. Briefly, each sample was placed centrally under a cylindrical probe (diameter 5 mm, height 40 mm) to determine the maximum force (N) when the probe penetrated the gel to a depth of 10 mm. The post-test, test, and pre-test speeds were set as 2.0 mm/s, 0.3 mm/s, and 2.0 mm/s, respectively, with a trigger type of 5 g auto force. The penetration force, which was the peak force needed to fracture the gels, was expressed as the gel strength [31]. All the experiments were performed 5 times and averaged.

### 2.10. Scanning Electron Microscopy Analyses

Scanning electron microscopy (SEM) was conducted following the procedure described by Gravelle [14] with minor modifications about different sample pretreatments and accelerating voltage. Gels were dipped in 2.5% glutaraldehyde for 48 h at 4 °C. Then, they were thoroughly dehydrated in a series of alcohols (30%, 50%, 70%, 90%, and 95% ethanol) for 15 min each and twice in 100% for 15 min. Thereafter, they were dried for 2 h. Briefly, SEM was performed with an S-4800 II FESEM scanning electron microscope (Hitachi High-Technologies Corporation, Tokyo, Japan). Specimens imaged by SEM were placed in the SEM chamber, placed under vacuum, and viewed under an accelerating voltage of 15 kV. The experiments were performed in triplicate.

### 2.11. Statistical Analysis

All experiments were carried out at least thrice, and the results were reported as averages and standard deviations of these measurements. Statistical analysis was performed using the SAS software (version 8.0) with one-way ANOVA and Duncan’s multiple range test (SAS Institute Inc., Cary, NC, USA). Differences with a *p* value of <0.05 were regarded as significant.

## 3. Results and Discussion

### 3.1. Characterization of Emulsions

#### 3.1.1. Drop Size

**Figure 1:**
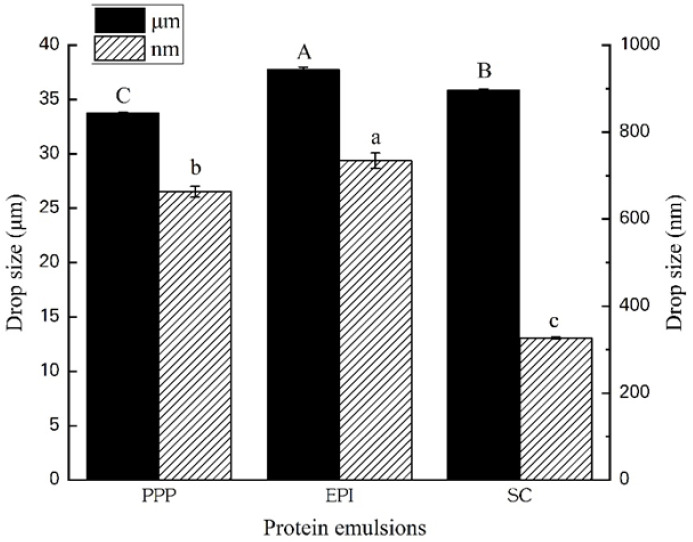
Changes of the average droplet diameters (*D*_4,3_) of protein emulsions after high-pressure homogenizing. For each kind of protein, the left column represents the droplet diameters of macro-emulsion, and the right column represents the droplet diameters of nano-emulsion. PPP: porcine plasma protein; EPI: egg-white protein isolate; SC: sodium caseinate. *n* = 5. Notes: Different letters (A–C) are significant (*p* < 0.05) of different droplet diameters of macro-emulsion and letters (a–c) are significant (*p* < 0.05) of the droplet diameters of nano-emulsion.

Droplet size is relevant to the physical property of emulsion-filled gel because droplet size determines the total contact area between the dispersed phase and the matrix [32]. Figure 1 reveals that high-pressure homogenizing on the nonmeat emulsions results in a significantly smaller mean droplet diameter than the non-homogenized samples (*p* < 0.05). Under high shear forces and high-frequency oscillation and cavitation, 15,000 psi homogenizing decreases the diameter of emulsion drop from macro- to nanoscale in the range of 324.4–734.5 nm for PPP, EPA, and SC emulsions, respectively. During homogenization, increasing the intensity or duration of energy could reduce the size of the emulsion drop [33]. Although our research has induced sufficient high pressure to create droplets as small as possible, a diameter in the 0–200 nm range is difficult to achieve because our emulsions were stabilized by biomacromolecule instead of small molecule emulsifier. Considering the linear shape molecule nature of MP, its emulsion cannot obtain a nanoscale droplet diameter, even under two times 15,000 psi high-pressure homogenizing. Other researchers have also found that 500 nm is the ultimate limit for the capability of microfluidizers to decrease the droplet size of macromolecule emulsion [34].

#### 3.1.2. Ability of Protein to Reduce the O/W Interfacial Tension

**Figure 2:**
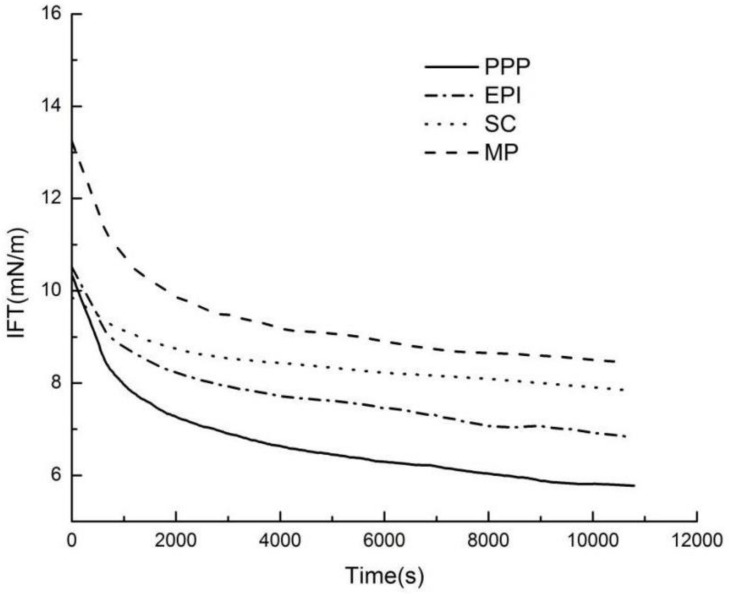
Dynamic interfacial tension of proteins at the O/W interface. PPP: porcine plasma protein; EPI: egg-white protein isolate; SC: sodium caseinate; MP: myofibrillar protein.

The determination of interfacial tension can observe the surficial properties on the O/W surface and the adsorption kinetics of the surface-active substance [35]. Interfacial tension curves of protein solutions containing MP, PPP, EPI, and SC at the same protein levels are shown in Figure 2. At the initial measurement stage, MP has a higher interfacial tension value than the three other nonmeat proteins. As time progresses, IFT values gradually decrease with adsorption time, and interfacial tension of all proteins significantly decreases at 10,000 s compared with that in the initial stage of the test. The fulfillment of protein O/W function can be associated with the adsorption at the interface and the conformation changes in the interface [36]. The final interfacial tension for MP is 8.47 mN/m, while that of the three other nonmeat proteins is less than 8.0 mN/m. Specifically, the value is 5.79 mN/m for PPP. Therefore, PPP, EPI, and SC have higher O/W surface activity than MP. SC molecules have relatively random and flexible structures [37] to achieve sufficient reduction in interfacial tension. Various constituents, such as globulins, ovalbumin, and ovotransferrin, contribute to the expression of surface-active properties of egg-white proteins [38]. The addition of bovine plasma proteins in the reduced-fat formulations improves the emulsifying stability of meat batter [39]. In this study, the interfacial activities of myofibrillar and three nonmeat proteins were compared for the first time. The results show that the ability of three nonmeat proteins to reduce the O/W interfacial tension is higher than that of MPs.

### 3.2. Viscosity of Emulsion-Filled Composite Sols

**Figure 3:**
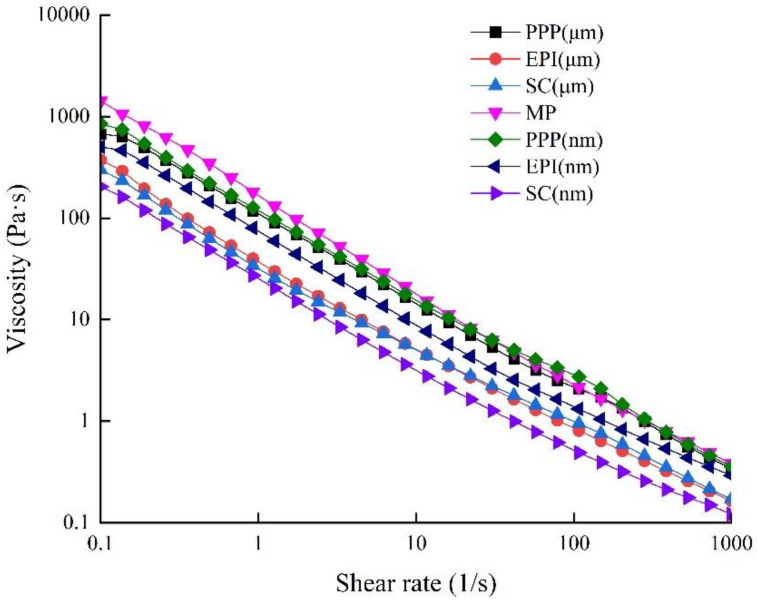
Viscosities of MP-emulsified soybean oil composite sols. PPP: porcine plasma protein; EPI: egg-white protein isolate; SC: sodium caseinate; MP: myofibrillar protein.

Viscosity can be used as a measure to test the internal friction and resistance to the flow of composite sols [40]. From previous studies [41], the viscosity of the emulsion-filled gel can be modified if whey protein isolate is pre-heated before emulsion followed by glucono-δ-lactone-induced gelation. So, it is valuable to explore more complex emulsion-filled gels in which pre-emulsified protein and continuous-phase protein are different. The viscosities of composite sols are shown in Figure 3. Compared with the viscosity value of MP alone, the initial viscosities of all the composite sols are significantly lower (*p* < 0.05) at a shear rate of 0.1 s^−1^. With the increase in shear rate, all the composite sols exhibit shear-thinning of viscosity values. Shear-thinning is one of the characteristic behaviors of many fluids polymer solutions and gels [42]. Once a shear rate is sufficient to overcome the Brownian motion and molecular collisions, less resistance to flow and lower viscosity will occur because the drop of emulsion is arranged orderly along the flow field [43,44]. Other researchers have also found that viscoelastic properties of dense gels containing droplets are very similar between filled gels of large and small droplets, and an increase in droplet size only causes minor changes in the rheological behavior of emulsion gel [45].

### 3.3. Heat Scanning Rheological Measurements

**Figure 4:**
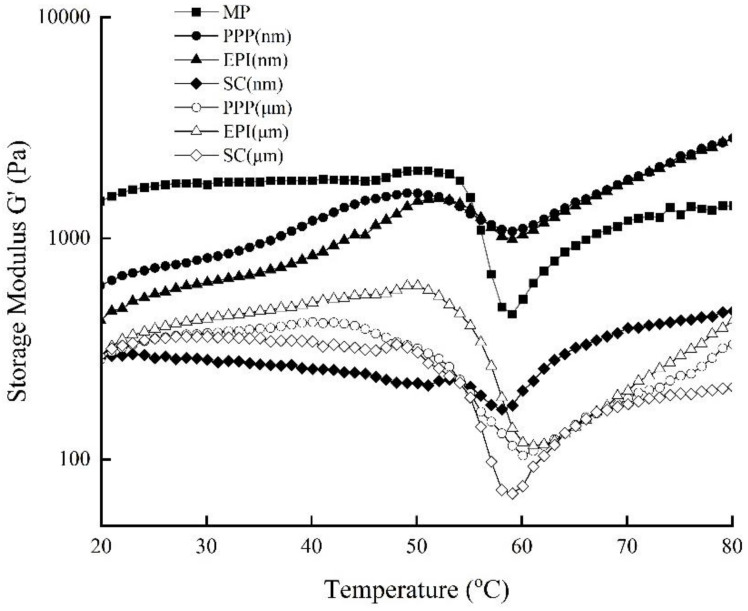
Heat scanning rheological dynamic storage modulus (G′) of MP-emulsified soybean oil composite sols. PPP: porcine plasma protein; EPI: egg-white protein isolate; SC: sodium caseinate; MP: myofibrillar protein.

Dynamic viscoelastic testing could reveal an insight into the interaction between proteins and fat globules during heating, which results in the formation and development of a composite viscoelastic gel network. Storage modulus (G′) was used to determine the energy stored resulting from elastic deformation, that is, additional deformation of the shape induced by an applied strain of the gel network [46]. Figure 4 shows that the storage modulus G′ of PPP, EPI, and SC emulsion composite sols increases during heating from 20 °C to 80 °C. Each kind of sol was made with two kinds of treatment groups of macro- and nano-emulsions, and MP macro-emulsion was regarded as the control.

In the composite sols, MP plays the critical role of building the gel matrix and is mainly responsible for the G′ formation. The G′ of MP emulsion sol increases slightly from 20 °C to 45 °C. Then, it increases up sharply to a maximum at approximately 50 °C. Thereafter, it decreases rapidly until 59 °C and rises steadily on subsequent heating to 80 °C. The increase in G′ at 4559 °C indicates the start of gelation, which is caused by the unfolding and association of heavy meromyosin. The temporary decline of G′ is probably due to the denaturation of light meromyosin [47]. In the subsequent heating to 80 °C, the formation of new bonds between MP is responsible for the new firm network [23].

The rheological properties of emulsion-filled gels could be influenced by the filler, gel matrix, and also the interactions between them. Actually, active and inactive fillers may leave distinct effects on the rheological behavior of emulsion gels [48]. When PPP and EA emulsions are incorporated into MP sol, the storage modulus of the two composite sol systems during heating obviously increases from 59 to 80 °C. However, when SC emulsion is incorporated into MP sol, the storage modulus development curve is gentler compared with those of PPP and EA during the same heating stage. The results indicate that PPP and EPI, other than SC, participate in the development of the MP gel networks and behave as active fillers. After the dispersing effect of high-pressure homogenizing, the oil droplets are fully covered with protein, which could protect them against coalescence and act as an anchor to interact with MP in the continuous-phase network [49]. According to previous reports [16], protein-coated emulsion droplets also work as active fillers in the heat-set whey protein matrix, which increases the elastic modulus of emulsion gel.

From the aspect of droplet size, using PPP and EPI nano-emulsions as fillers, other than SC, obtain higher final storage moduli, which are even more than that of MP alone. When SC nano-emulsion is mixed with MP, the final storage modulus is below the value of the MP alone. Under the same measurement parameter setting, the curve of SC nano-emulsion is different from those of other nano-emulsion composite sols. This difference may be due to the protein properties of SC. Specifically, it cannot form a gel by itself. In this case, SC acts as an inactive filler, which could weaken the network.

### 3.4. Strength of Emulsion-Filled Gels after Heating

**Figure 5:**
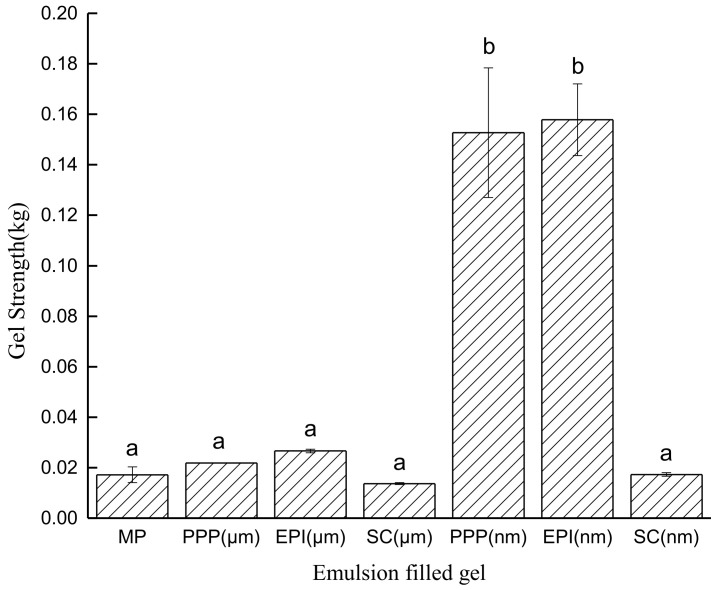
Gel strength of heat-induced MP-emulsified soybean oil composite gels. Gels were prepared from MP mixed with macro-emulsion and nano-emulsion of PPP, EPI, SC, and macro-emulsion of MP. PPP: porcine plasma protein; EPI: egg-white protein isolate; SC: sodium caseinate; MP: myofibrillar protein. Different letters in the same column means significant difference (*p* < 0.05), *n* = 5.

Gel strength, which is a common measure of gel quality, has always been used to determine the force required to break the gel and to characterize the mechanical properties of the gel. Figure 5 shows that MP macro-emulsion composite gels have a negligible effect on gel strength compared with the control MP group at equal protein levels. The nano-emulsion of PPP and EPI produces stronger gels than the inactive nano-filler, namely SC nano-emulsion (*p* < 0.05). This result corresponds well with the dynamic viscoelastic measurement (Figure 3), that is, the nano-emulsions of PPP and EPI behave as active fillers.

Similar to the rheological results, active fillers enhance the gel properties. The MP filled with nano-emulsion of PPP and EPI has nearly eight times gel strength compared with others. The formulation of emulsion gels plays an important role in determining their performance in texture modification [50]. Thus, the difference in the strength of emulsion-filled gels will be pronounced if the intermolecular cross-linking between interfacial and continuous-phase proteins mainly affects the gelation in addition to drop size.

### 3.5. Microstructure of Emulsion-Filled Gels after Heating

**Figure 6:**
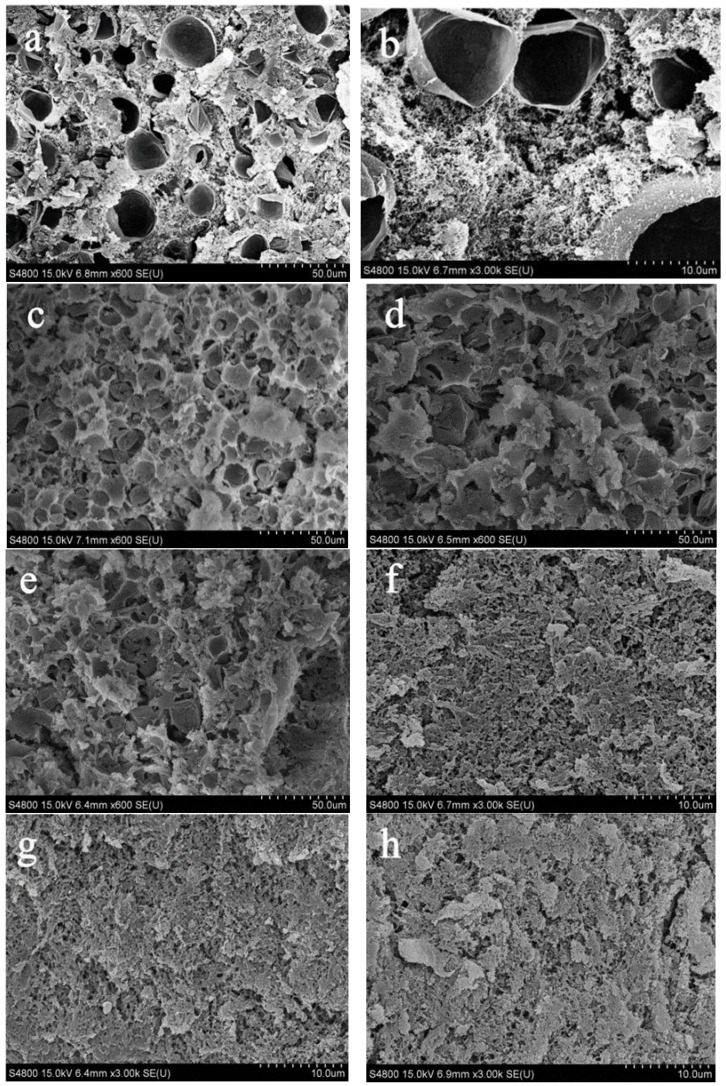
Microstructure of of different emulsion-filled gels. (**a**,**b**) MP gel, 600 and 3000 magnification, respectively; (**c**) PPP macro-emulsion composite gel, 600 magnification; (**d**) EPI macro-emulsion composite gel, 600 magnification; (**e**) SC macro-emulsion composite gel, 600 magnification; (**f**) PPP nano-emulsion composite gel, 3000 magnification; (**g**) EPI nano-emulsion composite gel, 3000 magnification; (**h**) SC nano-emulsion composite gel, 3000 magnification.

Figure 6 illustrates the obvious distinction in the size of fat globules in gels, which were made by mixing MP with different kinds of emulsions followed by heat-induced gelation. The microstructure of MP (Figure 6a) shows numerous pores with a size in the range of 20–25 μm, which is the largest in all samples (Figure 6a–h). The gels of macro-emulsion composites have rough and spongy appearances, with pores of a size mostly in the range of 10–15 μm. After high-pressure homogenizing, the pores of nano-emulsion composited gels are decreased and the gels show a high-density structure. Similar to the previous studies [51], high-pressure homogenizing reduced the drop size and increased the dispersion of emulsion, which further contributed to the mixed system being more uniform. Finally, a more stable three-dimensional network gel structure was formed. The filling effects of nano-sized emulsion droplets produced by homogenizing could explain this phenomenon. From the aspect of microstructure, the influencing factors of high-quality MP composites are the size of droplets and the type of pre-emulsion proteins.

## 4. Conclusions

The filling performance of emulsion droplet-coated protein to the heated-induced gel matrix was evaluated. PPP and EPI emulsion droplets were used as active fillers to increase the storage modulus and form a viscoelastic network. The active filling effects were supported by the results of gel strength and SEM. High-pressure homogenizing is an efficient method to conduct nano-emulsion and help the emulsion droplet-coated active protein enhance the filling effects on the gel matrix. Meanwhile, these results may provide new recommendations for regulating the texture of emulsion gels and selecting the correct pre-emulsified protein.

## Data Availability

Not applicable.
